# Normal Salt Solution by Hypodermoclysis, with Report of an Unusual Case

**Published:** 1901-11

**Authors:** G. A. Himmelsbach

**Affiliations:** Buffalo, N. Y., Assistant Attending Physician, Buffalo General Hospital.


### Normal Salt Solution by Hypodermoclysis, with Report of an Unusual Case.

By G. A. HIMMELSBACH, M. D., Buffalo, N. Y.,
Assistant Attending. Physician, Buffalo General Hospital.

THE therapeutic value of salt solution is now so well established, and its merits generally recognised, that it would seem time ill spent to review so commonplace a subject. However, I will venture to speak briefly of some of the methods of usage and their application in a few diseases, and conclude by reviewing the history of a very unique case which recently came under my observation, which I think you will agree was a valuable object lesson.

It is now quite generally conceded that its therapeutic value is not simply limited to the narrow confines of anemia, from blood loss or hemorrhage, as was first suggested, but today, diseases, acute and chronic, in great numbers, are all very much benefited by its judicious use. The methods usually adopted for its administration, for any given case, are, as a rule, one or all, of the following—namely, hypodermoclysis, intravenous and enteroclysis.

The method of administration is usually governed by the specific indications of the case in hand, and whether

Read before Buffalo Academy of Medicine, March, 1901.

immediate or less rapid results are called for. So much might be said of the particular advantages of each one of these methods, that a general review of them all would carry me beyond the limits of my memoranda. There are, however, a few points concerning the use of the saline solution which are common to all. I speak now with special reference as to its use subcutaneously.

I have referred to the first introduction of the salt solution, as a measure of especial use in hemorrhage from any cause. This has been in use for years and with the most satisfactory results. It is not simply of value in post operative cases, to restore the blood loss, but also in ante operative cases, like gunshot wounds, fractures, simple and compound, and other injuries, attended with shock and hemorrhage. It would seem at first thought, that this would be dangerous on account of increasing the blood pressure and thereby increasing the hemorrhage during the operation, but experience has proven the utility of a small or moderate hypodermoclysis of four to six ounces.

Salt solution subcutaneously has been used with advantage in cases of hemorrhage from gastric ulcer, as well as hemorrhage from the lung.

Dr. W. H. Thompson has had brilliant results in hemorrhage from typhoid ulcer. In infancy and children, its most valued use is perhaps in enterocolitis, attended with profuse watery discharges. Here the warm salt solution, given as high up enemata, not simply as a bowel wash to eliminate poison, but the absorption of a part of this fluid into the circulation and the stimulating effect of the heat, are very important. I would say, however, that in these cases, the combined use of salt solution, both by enemata and subcutaneously, is more efficacious.

Dawbarn, in 1892, was the first to call the attention of the profession to the use of saline solution in these cases. Since then it has been taken up and faithfully tried all over the world, and with results that show favorably, if they do not surpass any other therapeutic measure yet suggested for them. This treatment has been much in vogue in our own city for the last few years, and has been a common practice in the Children's Summer Hospitals. Dr. Sherman has reported to me personally the singular and unusually favorable termination of some of these cases by the use of the treatment which, in his judgment, would have proved fatal without it. He was the first to suggest to me a few years ago the advantage of small doses of the salt solution given subcutaneously, and he also called my attention to the importance of continuous peripheral massage from the very moment the fluid is introduced. This procedure is plain to you all, for it hastens absorption and prevents the formation of too much infiltration at the site of injection. Since his report, other writers have mentioned the same point. Kemp states that sloughing has occurred in the breasts, from a too large and too rapid injection of saline solution.[1]

Kemp has devoted much time studying the use of salt solution and has made experimental research on the lower animals, and has also contributed several very able papers during the past year, one in *Medical Record*, April 14, on Observations and Suggestions Concerning Hypodermoclysis, and a serial paper appearing in *Pediatrics*, October, November and December, 1900, on The Normal Saline Solution in Scarlatina Nephritis. To Kemp I am indebted for a few of the following interesting points, which he has had the pleasure of observing and reporting. He made a special study of the time taken by all three methods, from the moment of introduction till its appearance in the urine. To make his analyses more accurate, and his findings more positive, he added a small amount of a weak solution of potassium ferrocyanide to the saline solution, and the reappearance of this combination was discovered in the urine by means of the Prussian blue reaction with chloride of iron, in the following order: intravenous injection of ½ ounce of solution gave increased renal secretion and Prussian blue reaction in 1½ to 2 minutes. The urine drops were carefully counted and registered so as to avoid possible error. The quantity infused was so small as to have no appreciable effect on the general circulation. A subcutaneous injection of the same chemical combination caused an increased renal secretion, and its appearance in the urine in 3½ to 4 minutes. Infusion into the bowel of a small amount of saline solution, to which was added the same weak solution of potassium ferrocyanide, the urinary secretions were increased and the reaction appeared in the urine in 20 minutes.

These observations teach us two very important lessons: (1) the renal stimulant or the diuretic effect of the saline solution; (2) the length of time required by the use of this diuretic.

Of all the conditions in which the solution is used, it seems to me that essentially the effect in the system can be summarised under two heads, viz.: (1) increased volume in the vascular

1. See my report of case.

system; (2) its pronounced and positive diuretic effect. The second, however, does in a measure depend upon the first.

In the judgment of many who have given special thought to salt solution, its strong and paramount value is its power to open up one of the main avenues of excretion, *i. e.*, the kidneys, and in this respect it perhaps has no companion in its class.

All diseases productive of toxins in the circulation call for blood washing and blood cleansing. Coincident with these is the need for elimination or out pouring of poisons through renal stimulation. Edema following nephritis, whether acute, a sequelæ of any of the exanthemata, or chronic from cardiac or renal insufficiency, is materially reduced. Pulmonary edema or effusion in any of the serous sacs from pleurisy, peritonitis or pericarditis, have also been relieved. The unloading of the fluid and toxins in the blood from the arterial and venous circulation in turn, calls for an unloading of the fluids of these sacs, or the water soaked cellular tissue, into the lymphatic system, thence into the general circulation. Poisons in the blood, whether the products of tissue metabolism, causing uremia or toxemia, or alkaloidal poisons, such as belladonna or opium, are also known to be benefited.

A matter of great importance aside from the rapidity of its action, is the relatively small amounts that can be injected subcutaneously, which is followed by a very copious diuresis. Most writers now agree that the quantities formerly given in this manner, say from one to three pints, are entirely unnecessary, and that an equally specific effect can be produced upon the renal organs, causing them to pour out urine in amount many times greater than the quantity of fluid injected. This has its advantages over the other method, in that the time taken to do the operation is curtailed, which is of especial importance in a restless crying baby.

Lenhartz, *Deutsche Archives fur Klin. Med.*, advocates frequently repeated injections subcutaneously. From 2 to 6 ounces every three to four hours, has a better diuretic effect and cause less strain on the kidneys than a pint given several times daily.

Kemp also has observed ℥ iii.-iv. injected produce a secretion nearly five times that quantity within four hours, and blood and albumin disappear and casts diminish. It must be remembered that in certain diseases attended with low blood pressure or in shock, the lymphatic absorption of the fluid is very slow, and even small injections take considerable time for absorption.

Shakespeare in his report to the United States Government on Asiatic cholera, sums up the effect of salt solution on the system, which seems to cover all: (1) it supplies fluid to the anhydremic blood; (2) makes good the loss of heat; (3) it dilutes the thickened tissue fluids; (4) it stimulates the kidneys to action; (5) it aids in the elimination of toxic matters circulating in the blood and exercising their baneful effects on the tissues, particularly on the nervous system.

Simon Robbaski, aged 41 years; Polish. Lived in this country eight years; laborer; early history and family history negative; married man—wife and six children. Five years ago had gonorrhea. Well up to five months ago when he noticed that his work caused considerable dyspnea with precordial pains. His feet began to swell. Obliged to give up work and confined in his bed continuously for three months. He then managed to regain his feet again, and in his eagerness to earn a livelihood for his family, he attempted to work. He managed to keep up for two weeks when he was obliged to take to his bed again, from great epigastric and precordial pains, but particularly the former. The edema of his lower extremities was now as bad as ever and his physician sent him to the general hospital.

Upon entrance his appearance was that of one in great distress. His pains in the epigastric region were most marked, particularly upon the slightest physical exertion. His lower extremities and back were very edematous. Physical examination of the heart showed both mitral and aortic disease with dilatation. It was thought not hypertrophy, the apical impulse was very feeble. The urine examination—24 hours—450 c.c., dark amber; odor, normal; density, 1031—total solids, 32.50 gms. Indican increased; urea, 14.85 gms.; uric acid present; chlorides, normal; bile pigment, absent; sugar absent; albumen present.

Microscope,—numerous casts { Hyalin casts. Granular casts. Epithelial casts. }

Many leucocytes (pus), a few red blood cells, some small round cells.

With these findings, we realised the seriousness and really the hopelessness of the man's condition. However, we were determined to help him by temporarily relieving him, thereby prolonging his life.

His heart was supported with strychnin sulphate, combined with infusion of digitalis and acetate of potash and the occasional use of salt solution per rectum. Diet restricted to milk and absolute rest. These therapeutic measures were continued for a short time, with absolutely no apparent improvement. Diuretin, in gr. xv., combined with apocynum cannabinum, gr. xv., were now substituted, the latter being favorably known as the vegetable trochar in anasarcas conditions.

These two remedies were religiously administered, but with results that were anything but encouraging. The case was growing worse. Several times he had sinking spells, requiring prompt treatment by hypodermics. On the morning of June 26, the man's hands and face were cyanotic; arms and legs cold, respiration labored and pulseless at wrist; in fact, his condition was so desperate that Dr. Hopkins very wisely suggested a last resort measure, the use of normal saline solution subcutaneously.

His lungs were water logged; in fact, his face, arms and body generally were swollen almost beyond recognition. At 6 o'clock, June 26, Dr. McCarthy, the house physician, administered nearly a pint of normal saline solution in the left thorax, introducing the needle at a point about two inches below the left nipple, using a fountain syringe, the whole appliance, including the solution having been previously sterilised. The seance requiring about 15 minutes, peripheral massage was applied continuously. The exact measurement of the urinary secretion for the 24 hours preceding this test, was only 210 c.c., or 2-5 pint. At 6.35 p.m., or 20 minutes later, the patient voided 30 c.c., or 1 ounce of neutral urine with a sp. gr. of 1024. Beginning now, the experience for the next few days was the most phenomenal, I have ever witnessed. At 8 o'clock that evening, or in 1 hour and 25 minutes, he voided 450 c.c. of neutral urine, with a density of 1006—a drop of 18. At 8.30, or 30 minutes later, another 450 c.c.; at 9.30, 470 c.c.; at 10, 490 c.c.; at 10.40, 540 c.c.; 11.20, 570 c.c.; 11.45, 420 c.c.; 11.55, 150 c.c.; a total in exactly 6 hours of 3540 c.c., or 7 pints.

This unusual output did not terminate here, but continued with unceasing flow through the several days following. The total excretion of the first 24 hours following the administration of the salt solution was 6210 c.c., or exactly 6000 c.c. more than during the 24 hours preceding. The second 24 hours resulted in 6480 c.c., or 270 c.c. more than the first 24 hours. The third 24 hours, 4170 c.c. The fourth 24 hours, the quantity dropped to 1650 c.c.; or approximately a normal output. The total excretion of urine in the 72 hours following the hypodermoclysis was 16,860 c.c. or 33 2-5 pints. The man lost nearly 35 pounds in three days. So rapid was his loss in rotundity, that at the end of a week, he had shrunken to his real "flesh and bones" and one would have hardly known him to be the same man. It is interesting also to note the swiftness with which his kidneys acted. It will be observed from the foregoing table, that the quantity ranged from 150 c.c. in ten minutes to 570 c.c. in 40 minutes. During the first 24 hours, he was obliged to urinate 18 times, the second 24 hours, 36 times, and the third 24 hours, 26 times, or 80 times in 72 hours.

The specific gravity of the urine taken each time ranged from 1001 to 1010 (mostly 1002 to 1006.) The total quantity of urea in the three days was, first day, 7.32 gms.; second day, 8.75 gms. third day; 6.52 gms.; total, 22.59 gms.

It is also worthy of mention, that during all this drenching our patient partook of very little solid food, and his drink was comparatively trifling. Repeated examinations of his urine during the succeeding weeks of his illness showed a marked improvement. Up until June 28, casts were present, and from June 29 to July 2, four successive days, the microscope examination did not show the presence of casts. The albumin, however, remained stationery. The red blood cells, so plentiful at first, entirely disappeared and the leucocytes were comparatively few. On August 1, our patient returned to his home somewhat improved, but in anything but an enviable state of health.

From the time of his hypodermoclysis until the day of his departure, he had an area 1½ inches long by ¾ wide, at point of introduction of fluid, which had sloughed and was covered with what appeared to be a healthy dry scab. About the last of August the patient presented himself to the Erie County Hospital for treatment of this slough, and was cared for surgically by Dr. Clinton.

137 West Tupper Street.

---

### NOTES ON THE ACTION OF HEROIN AS COMPARED WITH THAT OF THE OTHER DERIVATIVES OF OPIUM.

In the *Montreal Medical Journal* for June, 1901, Gillies states that from his experience with the drug the following conclusions may be drawn: Heroin may be regarded as a valuable addition to our therapeutic agents. It is superior to morphin or codein in irritative coughs. In dyspneic conditions it is of special value. As an analgesic it is inferior to morphin, but heroin hydrochloride should have further trial before a definite statement is made in this respect. It is less toxic and therefore more safe than morphin and codein. He is as yet unable to state whether the habit is likely to be formed or not.—*Therapeutic Gazette.*

---

We should always bear in mind the fact that a fractured rib may penetrate into the lung, and, further, that a penetrating wound of the chest, if low down, may involve the peritoneal cavity. In a penetrating wound the outer layer of the pleura may be pierced and the viscera not damaged; the lung may be injured, the pericardium damaged, the heart penetrated or one of the great vessels may be entered.—DaCosta.

